# Controlling DNA Bundle Size and Spatial Arrangement in Self-assembled Arrays on Superhydrophobic Surface

**DOI:** 10.1007/s40820-014-0027-z

**Published:** 2014-12-30

**Authors:** Gabriele Ciasca, Massimiliano Papi, Valentina Palmieri, Michela Chiarpotto, Simone Di Claudio, Adele De Ninno, Ennio Giovine, Gaetano Campi, Annamaria Gerardino, Luca Businaro, Marco De Spirito

**Affiliations:** 1 Istituto di Fisica, Universitá Cattolica SC, L.go Francesco Vito 1, 00168 Rome, Italy; 2 Istituto di Fotonica e Nanotecnologie-CNR, Via Cineto Romano 42, 00156 Rome, Italy; 3 Institute of Crystallography-CNR, Via Salaria Km 29, 0016 Monterotondo, Rome Italy

**Keywords:** Superhydrophobicity, DNA array, Cassie state, Wenzel state, 1D nanostructures, Self-assembly

## Abstract

The use of superhydrophobic surfaces (SHSs) is now emerging as an attractive platform for the realization of one-dimensional (1D) nanostructures with potential applications in many nanotechnological and biotechnological fields. To this purpose, a strict control of the nanostructures size and their spatial arrangement is highly required. However, these parameters may be strongly dependent on the complex evaporation dynamics of the sessile droplet on the SHS. In this work, we investigated the effect of the evaporation dynamics on the size and the spatial arrangement of self-assembled 1D DNA bundles. Our results reveal that different arrangements and bundle size distributions may occur depending on droplet evaporation stage. These results contribute to elucidate the formation mechanism of 1D nanostructures on SHSs.

## Introduction

One-dimensional (1D) nanostructures such as polymeric, small molecules and inorganic types are currently under much investigation for their unique mechanical, optical, and electronic properties, as well as possible applications in design and realization of novel biomedical devices [1]. Over the past 20 years, most of the research efforts have been devoted to the synthesis of 1D nanostructures. To this purpose, many strategies based on top-down or bottom-up approaches have been successfully developed and applied [2]. Aside from the establishment of an effective synthesis route, the integration of 1D nanostructures into functional devices requires development of novel strategies to align such nanostructures in a parallel, scalable, and highly reproducible manner. A possible approach is based on the use of standard top-down lithographic techniques such as electron-beam lithography. However, this approach is often challenging, expensive, and time-consuming [1–4].

These limitations can be overcome by using superhydrophobic patterned surface. Historically, superhydrophobic surfaces have attracted much attention because of their self-cleaning properties that make them suitable for a variety of technological and industrial applications [5–15]. However, since a few years back, researchers have moved their interest to investigate whether superhydrophobic surfaces could be actively exploited to manipulate matter at the nanoscale level. This research effort led to design and test new devices with unexplored and attractive properties. Recently, the pioneering work of Su and co-workers demonstrated the possibility to exploit superhydrophobic surfaces to induce the self-assembly of strictly aligned organic filaments [16]. This result stimulated an intense research effort toward the realization of high-aspect ratio one-dimensional nanostructures with possible application in a variety of fields, including plasmonic materials (gold and silver nanoparticles), catalytic compounds, and DNA filaments [17–23].

**Fig. 1:**
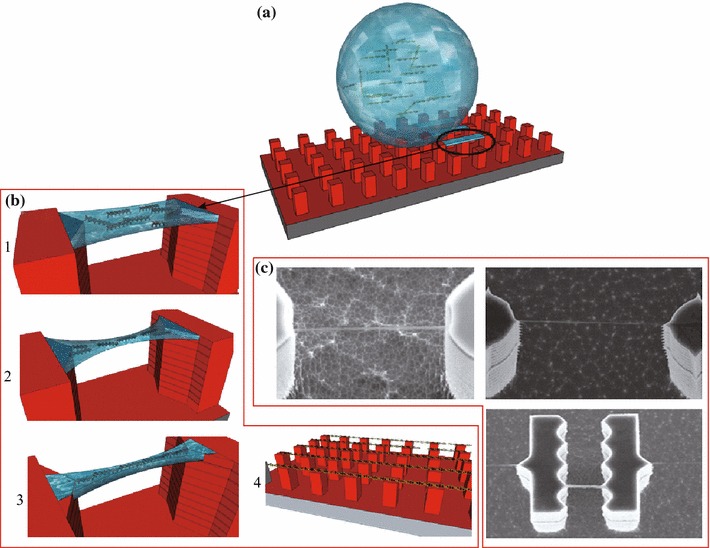
**a** Schematic view of the typical pillar-based superhydrophobic patterned surfaces, where a droplet containing DNA has been deposited. **b** Water capillaries between adjacent pillars (*step 1*); Water capillary evaporation pushes together the DNA strands (*step 2*); Formation of stable DNA bundles on the tips side (*step 3*); Highly ordered array of aligned 1D filaments (*step 4*). **c** SEM image of stretched DNA bundles on three different pillar geometries

The mechanism of the deposition of aligned and suspended filaments was described very recently for DNA bundles [19]. Briefly, a droplet containing DNA can be deposited onto a superhydrophobic surface wetted in the Cassie state. Under evaporation conditions, the retracting drop edge stretches DNA filaments along the de-wetting direction. While receding, the droplet forms capillaries which pin to the pillar protrusions allowing for the precise control of bundles position and orientation. For clarity, this mechanism is summarized in Fig. 1.

This self-assembling mechanism has the advantage of producing a remarkably uniform nanostructure size distribution under specific conditions. This feature is very important for device applications because well-aligned and uniform 1D nanostructures exhibit superior properties that have not been found in those of disordered arrangements [1]. However, the experimental conditions for this uniform size distribution have not been fully explored.

In order to explore these conditions, we investigated the size distribution and the spatial arrangement of self-assembled 1D DNA bundles obtained by droplet evaporation on SHSs. We revealed the presence of three spatial regions encompassing three different DNA bundle arrangements with different size distributions.

## Materials and Methods

DNA isolation was performed according to standard phenol/chloroform protocol as described elsewhere [19]. Superhydrophobic surfaces were fabricated directly on silicon substrate by electron-beam lithography (Vistec EPBG-5HR acceleration voltage: 100 keV) and inductively coupled plasma (ICP) Si etching [22–25]. A 400-nm-thick layer PMMA 950 K 9 % was spun on a Si wafer, exposed with a dose of 700 μC cm^−2^, and developed. A 30-nm-thick Cr film was then deposited by e-gun-assisted evaporation and lifted off. The device pattern was transferred on the substrate by ICP etching. After cleaning in Piranha solution (H_2_SO_4_/H_2_O_2_ = 3:1), both microstructured Si wafers were silanized with 10 % trimethylchlorosilane in toluene to impart the superhydrophobic behavior. This fabrication step was detailed discussed in Ref. [26].

Self-assembled arrays of DNA bundles were obtained by deposition of 5 µL droplets containing DNA at different concentrations. After deposition, the superhydrophobic surface was tilted by 10° and the droplet was let to completely evaporate at room temperature. In this configuration, the sample solution droplet slowly slips downward along the tilted surface, allowing for the formation of highly ordered arrays of aligned 1D DNA filaments. The superhydrophobic surfaces were characterized by scanning electron microscopy (SEM, Zeiss Supra 120) after deposition of a 6-nm-thin Cr layer in order to avoid charging effects and to protect DNA filaments from e-beam damage [27, 28].

## Results and Discussion

**Fig. 2:**
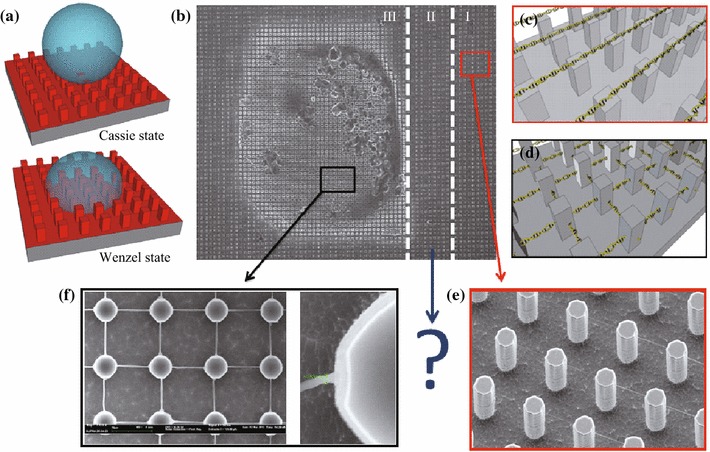
**a** Schematic view of a water droplet on a superhydrophobic surface wetted in the Cassie state (*above*) and in the Wenzel state (*below*). **b** Typical DNA stain on a superhydrophobic surface due to the Cassie-to-Wenzel transition. **c** Schematic view of the typical DNA bundle arrangement occurring in region I; **d** Schematic view of the DNA bundle array formed in region III. **e** SEM image of the typical DNA arrangement in region I; **f** SEM image of the peculiar DNA arrangement that might be obtained inside the DNA stain in particular experimental conditions [20]

The behavior of water drops deposited on superhydrophobic surfaces under evaporation conditions consists of two states: either the drop sits on the tops of the pillars (Cassie state), or it sinks inside the texture (Wenzel state). In the first state, the drop evaporates sitting stably onto the pillars. However, as the drop recedes, a transition from the Cassie state to the Wenzel state may occur [5–15]. This process is schematically displayed in Fig. 2a. After droplet evaporation, a circular stain is usually observed at the bottom of the patterned surface. The typical stain obtained after evaporation of a 5 μL DNA solution droplet (150 ng μL^−1^) is shown in Fig. 2b. For clarity, three different regions were highlighted on the surface depending on the distance from the stain (Fig. 2b).

In region I, sufficiently distant from the DNA stain, the evaporation dynamics of a droplet in the Cassie State leads to the formation of highly ordered arrays of stretched DNA strands. In this region, DNA strands are strictly suspended on the top of the pillars as previously demonstrated in Ref. [19]. A schematic view and a SEM image of the typical DNA bundles arrangement is shown in Fig. 2c, e, respectively. Conversely, within the DNA stain (region III), the ordered arrangement was usually not present and an unstructured solute deposit was observed instead. The shape and the size of the solute stain may depend on many parameters such as solute concentration, temperature, and pillars shape as recently demonstrated by Dicuangco et al. [29]. However, in particular experimental conditions, an ordered arrangement may be observed. In these conditions, DNA may also form bundles pinned at different heights to the body of the pillars suggesting that a tridimensional order can be obtained (Fig. 2d, f) [20].

**Fig. 3:**
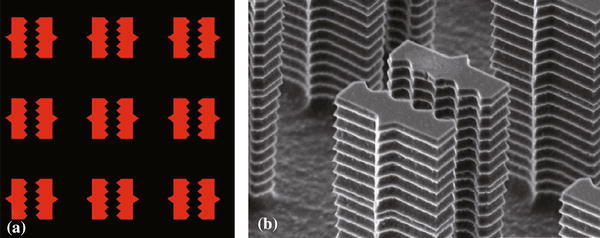
**a** Schematic view of adopted superhydrophobic surface; **b** Tilted SEM image of the microstructured superhydrophobic surface

Moreover, in region I, DNA bundles show a narrow size distribution centered around 20 nm, whereas in region III the average bundle size is about 200 nm. A SEM image of bundles in this region is shown in Fig. 2e. Further details on the bundles behavior in these two limit cases were discussed in Refs. [19] and [20]. However, the bundles behavior in the intermediate region between region I and III (region II), which is endowed with a higher degree of complexity, has not been assessed yet. To address this issue, particular pillar geometry, namely a saw-shaped pillar, was micro-fabricated. The schematic view of the adopted SHS is shown in Fig. 3a. Each pillar is 10 μm in side, 14 μm pitch, and the distance between the two halves of the same pillar ranges between 4 and 2 μm (Fig. 3a). The triangular-shaped protrusions are molded to pin DNA strands both inside and outside the saw-shaped pillars. A SEM image of the SHS is shown in Fig. 3b. The described features make the contemporaneous investigation of multiple length scales possible as clarified in the following.

**Fig. 4:**
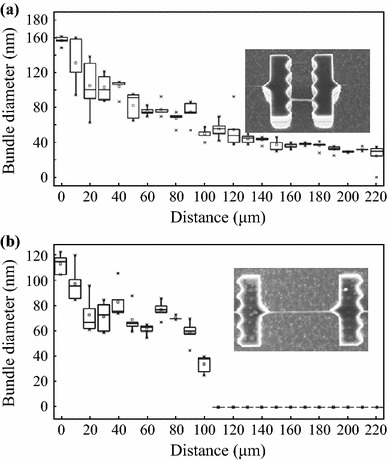
**a** The average diameter of the bundles pinned inside the saw-shaped pillars in region II as a function of the distance from the DNA stain shown in Fig. 2b. **b** The average diameter of the bundles pinned outside the saw-shaped pillars as a function of the distance from the stain. A drop volume of 5 μL and a DNA concentration of 150 ng μL^-1^ was used

Figure 4a shows the average diameter of bundles pinned inside the saw-shaped pillars as a function of the distance from the stain in region II. Each value is the average of 15 measurements acquired on three different devices. In the closest proximity to the DNA stain, the bundle diameter is ~160 nm. This value is of the same order of magnitude than that measured within the stain. An exponential decrease of the average bundle diameter down to ~30 nm can be observed across 170 μm from the stain. Since a 6-nm Cr layer against e-beam strand damage has to be considered, the resulting average bundle diameter at the plateau is ~20 nm, which is consistent with that measured in region III [20].

**Fig. 5:**
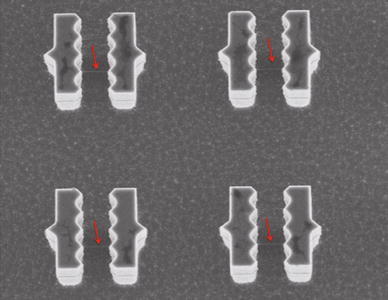
SEM images of DNA far from the stain where DNA bundles pinned outside the *saw-shaped pillars* (undetectable) and inside the *saw-shaped pillars* (detectable)

**Fig. 6:**
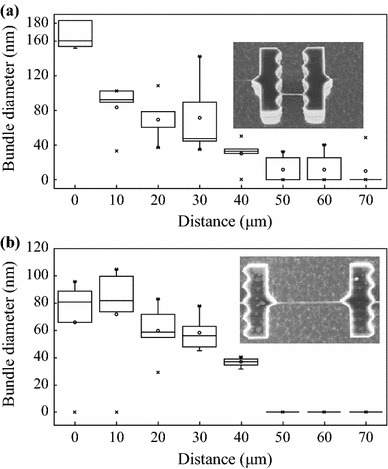
**a** The average diameter of the bundle pinned inside the saw-shaped pillars in region II as a function of the distance from the DNA stain shown in Fig. 2b. **b** The average diameter of the bundle pinned outside the saw-shaped pillars as a function of the distance from the stain. A drop volume of 5 μL and a DNA concentration of 50 ng μL^-1^ was used

Figure 4b displays the average diameter of the bundles pinned outside the saw-shaped pillars as a function of the distance from the stain in region II. It is clear that the bundles are significantly thinner than those pinned inside the saw-shaped pillars. In the closest proximity to the DNA stain, the bundle size is ~110 nm, which is the same order of magnitude of the diameters distribution that might be observed within the stain. Then, the average bundle diameter decreases monotonously up to reach about 40 nm at 100 microns far from the stain. From this point, DNA bundles are no longer detected inside the saw-shaped pillars as shown in Fig. 5. This may be due to the fact that bundles pinned outside the saw-shaped pillars are significantly longer and thinner than those pinned inside the pillars and therefore lack the necessary structural support Fig. 6.

Furthermore, we also investigated whether the concentration of the DNA solution plays a significant role in regulating the bundles size. To this purpose, 5 μL of 50 ng μL^−1^ genomic DNA solution was deposited on a superhydrophobic surface wetted in the Cassie state. Referencing the bundles pinned outside the saw-shaped pillars, the average bundle diameter dependency on the distance from the stain is shown in Fig. 6a. Like the higher concentrated DNA solution, the bundle diameter is ~160 nm in the closest proximity to the DNA stain. Then, it decreases exponentially up to reach a plateau of ~15 nm at 60 μm far from the stain. Referencing the bundles pinned outside the saw-shaped pillars (Fig. 6b), the average diameter is ~80 nm in the closest proximity to the DNA stain and decreases monotonously up to 40 nm, like the more concentrated ones. DNA bundles can no longer be detected at 40 microns far from the stain.

## Conclusion

In this work, we investigated the size distribution and the spatial arrangement of self-assembled DNA bundles formed by droplet evaporation on superhydrophobic surfaces. It was found that both DNA bundle size and its arrangement depended mainly on the droplet evaporation stage. When the droplet was in the Cassie state, ordered array of ~20 nm DNA filaments was formed. As the evaporation proceeded, a region where the bundle size increased from few tens to few hundreds of nanometers was observed, and the scale of this region depended strongly on the initial DNA concentration. The length of the region is ~170 μm at high concentration of 150 ng μL^−1^ and ~40 μm at low concentration of 50 ng μL^−1^. Moreover, the bundle size also depended on the distance between adjacent pillars: the larger the distance, the thinner the DNA bundles. In the last phase of the evaporation process, the continuous shrinking of the droplet induced a state transition from Cassie to Wenzel and resulted in a disordered or square-shaped DNA arrangement. Taken together, these results show that the complex evaporation dynamics of a water droplet onto a superhydrophobic surface must be carefully considered when using these surfaces to obtain a self-assembled array of aligned 1D nanostructures. Moreover, the data presented here have the potential to provide a great advantage for those applications where a precise control of the bundle sizes is required.
